# Record-matching of STR profiles with fragmentary genomic SNP data

**DOI:** 10.1038/s41431-023-01430-9

**Published:** 2023-08-11

**Authors:** Jaehee Kim, Noah A. Rosenberg

**Affiliations:** 1 Department of Computational Biology, Cornell University, Ithaca, NY 14853 USA; 2 Department of Biology, Stanford University, Stanford, CA 94305 USA

**Keywords:** Population genetics, Computational biology and bioinformatics

## Abstract

In many forensic settings, identity of a DNA sample is sought from poor-quality DNA, for which the typical STR loci tabulated in forensic databases are not possible to reliably genotype. Genome-wide SNPs, however, can potentially be genotyped from such samples via next-generation sequencing, so that queries can in principle compare SNP genotypes from DNA samples of interest to STR genotype profiles that represent proposed matches. We use genetic record-matching to evaluate the possibility of testing SNP profiles obtained from poor-quality DNA samples to identify exact and relatedness matches to STR profiles. Using simulations based on whole-genome sequences, we show that in some settings, similar match accuracies to those seen with full coverage of the genome are obtained by genetic record-matching for SNP data that represent 5–10% genomic coverage. Thus, if even a fraction of random genomic SNPs can be genotyped by next-generation sequencing, then the potential may exist to test the resulting genotype profiles for matches to profiles consisting exclusively of nonoverlapping STR loci. The result has implications in relation to criminal justice, mass disasters, missing-person cases, studies of ancient DNA, and genomic privacy.

## Introduction

In forensic genetics, the identity of a DNA profile is often sought from a biological sample with poor DNA quality, for which standard molecular techniques used with high-quality samples are unlikely to successfully produce genotypes. When the sample originates from trace sources such as burned, degraded, or ancient materials, only limited portions of the original genome might remain in the sample.

**Fig. 1:**
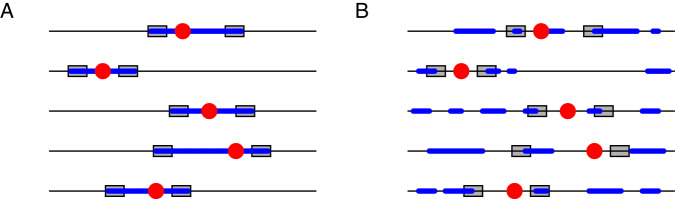
Genotyping of fragmented DNA might fail to amplify STRs, but it can amplify SNPs in the neighborhood of STRs. Each row depicts a chromosome, with an STR locus in red. The blue regions represent genotyped segments. **A** In high-quality DNA samples, STRs are genotyped by amplifying regions bracketed by PCR primers, depicted as gray boxes. **B** In low-quality DNA samples, PCR primers might not amplify, but some of the SNPs near an STR can be genotyped.

Routine genotyping of short-tandem-repeat loci (STRs) assumes that high-quality DNA samples contain DNA fragments in long sections of sequence. Hence, in a high-quality sample, the polymerase chain reaction can amplify the fragment that contains the entire section of DNA that lies between a specified pair of primer sequences [e.g., [1]]. The amplification relies on the inclusion of both the primers and the fragment connecting them—which contains an STR region—in the DNA sample (Fig. 1A).

For degraded DNA samples, however, standard STR genotyping procedures can be unlikely to succeed [2–4]. DNA fragments in the biological sample might be short and scattered, so that it is improbable that both primers and the DNA between them are present to be amplified. Nevertheless, although STR genotyping might fail, next-generation sequencing might be capable of producing genotypes of the available fragments (Fig. 1B). Genetic information might be possible to extract, and in particular, genotypes might be possible to generate for some of the single-nucleotide-polymorphism (SNP) sites in the genome [e.g., [5–12]].

With next-generation sequencing of fragmentary materials, no particular genomic site can be reliably expected to appear in the genotype data. In particular, the STR loci that underlie standard forensic databases [13–15]—and that are genotyped by amplifying specific genomic sites—are unlikely to be obtained from the sample of interest, nor is any specific target set of SNPs. Thus, when an investigator seeks to query an unknown degraded sample for a match to STR genotypes of a known individual or relative, or to search an STR profile database for a match, the fragmentary genotypes represent different and apparently incommensurable genetic loci from those available for potential matches.

Is it possible to identify genetic matches between a fragmentary SNP genotype profile from a degraded DNA sample and the genotypes of a nonoverlapping set of STRs? In a technique termed “genetic record-matching,” we have recently shown that, owing to genotypic correlations between STRs and their neighboring SNPs, it is frequently possible to identify matches between pairs of profiles, when one member of the pair is a SNP profile and the other is a forensic STR profile [16]. Furthermore, it can often be determined that two profiles, one containing genome-wide SNPs and the other with forensic STRs, represent close relatives [17].

Our calculations, however, have made use of genome-wide SNP datasets with high genotyping quality, with high genomic coverage around each forensic STR locus. What if the SNP data were instead fragmentary, in the manner expected for degraded DNA and fragmented genotyping? This problem of record-matching between STR profiles and fragmentary SNP profiles represents any of several possible scenarios: matching the SNP profile of a degraded crime-scene sample to the STR profile of a specific known suspect, querying a degraded crime-scene SNP profile against a database of STR profiles, matching the SNP profile of an ancient DNA sample to specific STR profiles of possible living relatives, matching the SNP profile of a degraded DNA sample in a missing-persons or mass-disasters case to STR genotypes from known missing persons or their relatives, or querying it against an STR database of many potential candidates.

Here, we consider genetic record-matching between STRs and fragmentary SNP data. We assess many genomic coverage levels, examining scenarios in which the hypothesis is that a SNP profile and an STR profile originate from the same person, from a parent–offspring pair, or from siblings.

## Materials and methods

### Dataset

We examine two datasets containing both SNP and STR genotypes. First, the Human Genome Diversity Panel dataset (HGDP), as studied by [16] and [17], contains unphased genotypes at 642,563 SNPs and 17 Codis STRs in 872 individuals from 52 populations—the 13 original Codis loci and 4 in the expanded set.

The second dataset is a phased reference SNP–STR haplotype panel of Saini et al. [18] from the 1000 Genomes Project phase 3 [19, 20] with high-quality SNP genotypes obtained from whole-genome sequencing. The 1000 Genomes dataset contains 2504 individuals from 26 populations, with data at 11 of the 13 original Codis core loci and all 7 expanded Codis core loci [15], and genomic data at 27,185,239 SNPs.

Tables S1 and S2 compare the HGDP and 1000 Genomes datasets. The 1000 Genomes has higher SNP density in the neighborhood of each Codis STR than does the HGDP, with an average of ~11,000 SNPs in a 1-Mb window centered at an STR locus in the 1000 Genomes compared to ~275 SNPs for HGDP.

Our previous record-matching studies used the HGDP dataset [16, 17]. Using a larger number of SNPs, Saini et al. [18] showed that genotype imputation accuracies at Codis STRs from neighboring SNPs are slightly higher when using denser 1000 Genomes data (see their Table S2). As record-matching relies on imputation, we expect that the 1000 Genomes will also produce higher record-matching accuracies than the HGDP.

To enable comparisons of record-matching accuracies in the 1000 Genomes and HGDP datasets, we focus on the 15 Codis loci present in both datasets (Table S2)—11 from the original Codis STRs and 4 from the expanded Codis STRs—treating all individuals within a dataset as members of a shared population.

### Genetic record-matching

We examine familial relationships between a pair of individuals, one from an STR dataset and the other from a SNP dataset typed at specified genomic sequencing coverage.

#### The relatedness match score

We follow Kim et al. [17] in computing match scores between profile pairs. For individual *i*, let the diploid genotype at STR locus *ℓ* be *R*_*iℓ*_ and let the diploid set of unphased genotypes at the neighboring SNP loci be *S*_*iℓ*_. Considering *L* STR loci of individual *i*, *R*_*i*_ = {*R*_*i*1_*, R*_*i*2_, *…*, *R*_*iL*_} is the STR profile from the STR dataset; *S*_*i*_ = {*S*_*i*1_*, S*_*i*2_, ..., *S*_*iL*_} is the SNP profile from the SNP dataset.

With no inbreeding, **∆** = (∆_0_, ∆_1_, ∆_2_) summarizes the relationship of two diploid individuals, giving probabilities of three identity states *C*_0_*, C*_1_*, C*_2_ [21]. Each *C*_*k*_ represents a configuration in which, for their unordered diploid genotypes at an autosomal locus, exactly *k* alleles are shared identically by descent. Notation (∆_0_, ∆_1_, ∆_2_) follows Kim et al. [17]; Jacquard’s (∆_9_, ∆_8_, ∆_7_) or Cotterman’s (*k*_0_, 2*k*_1_*, k*_2_) can also be used.

We test a specified relatedness hypothesis **∆**_test_ between individual *A* with STR profile *R*_*A*_ and individual *B* with SNP profile *S*_*B*_ against a null model in which the individuals are unrelated. The test uses the log-likelihood-ratio relatedness match score comparing alternative and null hypotheses [17], or

1 $$\lambda \left( {R_A,S_B} \right) = \mathop {\sum}\limits_{\ell = 1} ^L {\left[ {\ln \left[ {{\Bbb P}\left( {R_{A\ell }\left| {S_{B\ell },\,{{{\mathbf{\Delta}}}} _{{{{{{{{\mathrm{test}}}}}}}}}} \right.} \right)} \right] - \ln \left[ {{\Bbb P}\left( {R_{A\ell }} \right)} \right]} \right]},$$

assuming independence of the STR loci (linkage equilibrium). We decompose P(*R*_*Aℓ*_ | *S*_*Bℓ*_, **∆**_test_) over possible values of *R*_*Bℓ*_, the STR profile of individual *B* at locus *ℓ*:

2 $${\Bbb P}\left( {R_{A\ell }\left| {S_{B\ell },\,{{{\mathbf{\Delta}}}} _{{{{{{{{\mathrm{test}}}}}}}}}} \right.} \right) = \mathop {\sum}\limits_{R_{B\ell } \in {{{{{{{\mathcal{R}}}}}}}}_\ell } {{\Bbb P}\left( {R_{A\ell }\left| {R_{B\ell },{{{\mathbf{\Delta}}}} _{{{{{{{{\mathrm{test}}}}}}}}}} \right.} \right){\Bbb P}\left( {R_{B\ell }\left| {S_{B\ell }} \right.} \right)}.$$

${{{{{{{{\mathcal{R}}}}}}}}_\ell }$ denotes the set of possible genotypes at locus *ℓ*. P(*R*_*Aℓ*_ | *R*_*Bℓ*_, **∆**_test_) is the probability of the observed STR genotype of individual *A* at locus *ℓ* conditional on a possible STR genotype of individual *B* at locus *ℓ* and the assumed relatedness hypothesis [21]. Evaluation of P(*R*_*Aℓ*_ | *R*_*Bℓ*_, **∆**_test_) follows Kim et al. [17].

P(*R*_*Bℓ*_ | *S*_*Bℓ*_) is the probability of possible STR genotypes of individual *B* at an STR locus *ℓ* conditional on the observed SNP profile surrounding STR locus *ℓ* of individual *B*. We use BEAGLE and a phased SNP–STR haplotype reference to impute and obtain probabilities of unobserved genotypes at the STR locus *ℓ*; BEAGLE details appear in Section S1.1. We consider three relationship hypotheses **∆**_true_ for *R*_*A*_ and *S*_*B*_: same individual, **∆**_true_ = (0, 0, 1); parent–offspring, **∆**_true_ = (0, 1, 0); and sibling pairs, ${{{\mathbf{\Delta}}}} _{{{{{{{{\mathrm{true}}}}}}}}} = \big( {\frac{1}{4},\frac{1}{2},\frac{1}{4}} \big)$.

#### Prior and posterior odds

We report some of our results in terms of prior and posterior odds. Consider two hypotheses,

*H*_0_: *A* with STR profile *R*_*A*_ and *B* with SNP profile *S*_*B*_ are unrelated;

*H*_1_: *A* with STR profile *R*_*A*_ and *B* with SNP profile *S*_*B*_ are related with relationship **∆**.

Following Edge et al. [16], using Eq. (1), we can simplify the posterior odds for hypothesis *H*_1_:

3 $$\frac{{P\left( {\left. {H_1} \right|R_A,S_B} \right)}}{{P\left( {\left. {H_0} \right|R_A,S_B} \right)}} = \frac{{P\left( {\left. {R_A} \right|H_1,S_B} \right)}}{{P\left( {\left. {R_A} \right|H_0,S_B} \right)}}\cdot \frac{{P\left( {\left. {H_1} \right|S_B} \right)}}{{P\left( {\left. {H_0} \right|S_B} \right)}} = e^{\lambda \left( {R_A,S_B} \right)}\cdot \frac{{{{{{{{{\mathrm{P}}}}}}}}\left( {H_1} \right)}}{{{{{{{{{\mathrm{P}}}}}}}}\left( {H_0} \right)}}.$$

The posterior odds for *H*_1_ is the product of the likelihood ratio P(*R*_*A*_ | *H*_1_*, S*_*B*_)*/*P(*R*_*A*_ | *H*_0_*, S*_*B*_) = $e^{\lambda \left( {R_A,S_B} \right)}$ and the prior odds for *H*_1_, P(*H*_1_)*/*P(*H*_0_). It is simplified in terms of the match score *λ*(*R*_*A*_*, S*_*B*_) (Eq. (1)).

#### Match assignment

We assigned matches from pairwise match scores as in Kim et al. [17]. For an STR dataset with *I*_*R*_ individuals and a SNP dataset with *I*_*S*_ individuals, we evaluated the match score under a test hypothesis **∆**_test_ (Eq. (1)) for all pairs of individuals, one with an STR profile and the other with a SNP profile. Here, *I*_*R*_=*I*_*S*_=*I*.

We constructed an *I* × *I* match-score matrix *M*, where for all *j, k* in the set [*I*] = {1, 2*, …, I*}, *M*_*jk*_ = *λ*(*R*_*j*_*, S*_*k*_) is the entry for STR profile *R*_*j*_ from individual *j* and SNP profile *S*_*k*_ for individual *k*. From matrix *M*, we assigned matches by one of four schemes [16, 17] (Section S1.2). Under one-to-one or one-to-many matching (with a query SNP profile or query STR profile), record-matching accuracy is defined as the fraction of pairs matched correctly among *I* true matches. In needle-in-haystack matching, accuracy is defined as the proportion of true matches with greater match scores than the largest score across all non-matching pairs.

### Pedigrees

To investigate familial record-matching, following Kim et al. [17], we simulated random pedigrees from data on unrelated individuals. Details of the pedigree simulation appear in Section S1.3.

### Record-matching with HGDP and 1000 Genomes

We first evaluated HGDP and 1000 Genomes record-matching accuracies with the 15 Codis loci described in Section “Dataset.” Following Edge et al. [16] and Kim et al. [17], we partitioned the data into disjoint training and test sets, with 75% of the individuals in the training set. For all three scenarios (same-individual, parent–offspring, sib-pair), we generated 100 random partitions (Section S1.4). We phased HGDP training sets using BEAGLE to obtain SNP–STR haplotypes that we used as a reference. Next, to estimate the unobserved STR genotype probabilities (P(*R*_*Bℓ*_ | *S*_*Bℓ*_) in Eq. (2)), we again used BEAGLE for imputing STR genotypes from test-set SNP profiles with the phased SNP–STR haplotype reference panel from the training set (Sections S1.1, S1.5).

For all three relatedness scenarios, for each of the 100 partitions, we constructed the match-score matrix from the test set and assessed record-matching accuracies for the four matching schemes (Section “Match assignment”). Median, minimum, and maximum record-matching accuracies of the 100 replicates using the HGDP dataset appear in Table S3; values with 1000 Genomes appear in Table 1 when **∆**_true_ = **∆**_test_, and in Table S4 when **∆**_true_ ≠ **∆**_test_. For the median, we used the lesser choice when the number of unique values was even.

### Simulation of fragmentary genomic SNP data

To generate random fragmentary genomic SNPs for the 1000 Genomes, for each relatedness scenario, we selected a partition corresponding to the median one-to-one match accuracy with **∆**_true_ = **∆**_test_ (Section “Record-matching with HGDP and 1000 Genomes,” Table 1). The match accuracy varies discretely across partitions; when multiple partitions all produce the median value, we picked one at random. Because the HGDP dataset is much smaller than the 1000 Genomes dataset, we conducted simulations of fragmentary SNP data for the 1000 Genomes only.

Under each choice of relatedness, from the full-coverage SNP profiles in the median-accuracy test set, we simulated fragmentary SNP data. For the same-individual scenario, this test set has 626 individuals; for the parent–offspring and the sib-pair scenarios, it has 313, one for each test-set pedigree described in Section S1.4.

**Fig. 2:**
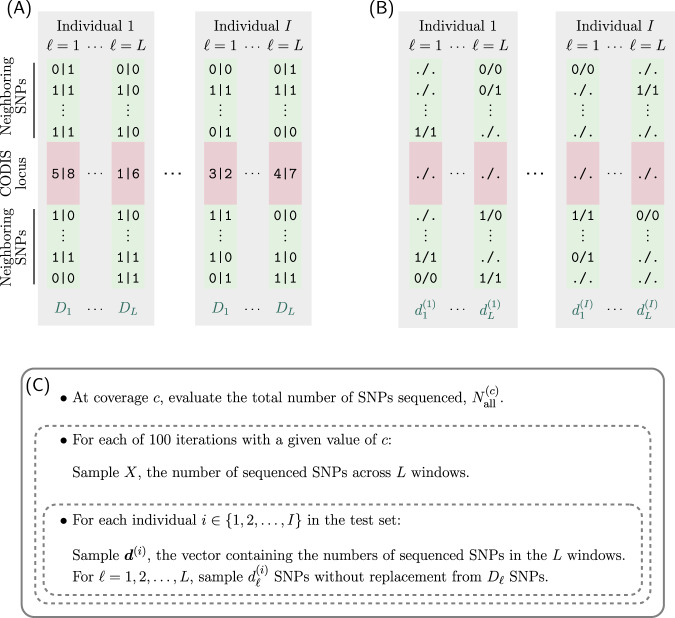
Schematic for simulating fragmentary SNP datasets for the individuals in the test set. **A** An example of the SNPs in a 1-Mb window (green) of a Codis locus (red) in two specific individuals. We denote the total number of SNPs in the whole genome with full coverage (*c* = 1) by *N*_all_ = 27,185,239. *D*_*ℓ*_ (*ℓ* = 1, 2*, ..., L*) indicates the number of SNPs in the 1-Mb window of the *ℓ*th Codis locus, and $N_{win} = \mathop {\sum}\nolimits_{\ell = 1}^L {D_\ell = 161,968}$ represents the number of SNPs in all *L* 1-Mb windows (Table S2). The symbol ‘|’ indicates phased genotypes. **B** The simulated set of fragmentary SNPs for the individuals in (**A**). The symbol ‘*/* ’ indicates unphased genotypes. **C** The simulation pipeline for generating simulated fragmentary SNPs from the 1000 Genomes dataset. For a given sequencing coverage *c*, the total number of SNPs sequenced from the whole genome is $N_{{{{{{{{\mathrm{all}}}}}}}}}^{\left( c \right)} = \left[ {N_{{{{{{{{\mathrm{all}}}}}}}}}c} \right]$. Given *c*, we repeat the following procedure 100 times to generate 100 random sets of fragmentary SNPs. We first sample *X*, the number of sequenced SNPs in *L* 1-Mb windows combined, from a binomial distribution with parameters $N_{{{{{{{{\mathrm{all}}}}}}}}}^{\left( c \right)}\,{{{{{{{\mathrm{and}}}}}}}}\,f = N_{{{{{{{{\mathrm{win}}}}}}}}}/N_{{{{{{{{\mathrm{all}}}}}}}}} \approx 0.006$. Using the sampled value of *X*, for each test individual *i* (*i* = 1, 2, ..., *I*), we generate random sets of sequenced SNPs in the 1-Mb windows by first sampling individual-specific ${{{\boldsymbol{d}}}}^{( i )} = ( {d_1^{( i )},\,d_2^{( i )},...,\,d_L^{( i )}} )$—the vector of numbers of sequenced SNPs from each of the *L* windows—from a multinomial distribution with parameters *X* and (*D*_1_*/N*_win_*, D*_2_*/N*_win_, ..., *D*_*L*_*/N*_win_). For each *ℓ* (*ℓ* = 1, 2, ..., *L*), we then sample $d_{\ell}^{(i)}$ SNPs uniformly at random without replacement from the *D*_*ℓ*_ SNPs of the full-coverage set.

Among *N*_all_ = 27,185,239 SNPs in the 1000 Genomes, the number in the 1-Mb windows around the *L* = 15 STR loci was *N*_win_ = 161,968 (Table S2). We considered 30 values of genomic sequencing coverage *c*: {0.004, 0.006, 0.008, 0.01, 0.02, ..., 0.19, 0.2, 0.3, ..., 0.8, 0.9}. With the partition into training and test sets fixed, for each *c*, we generated 100 random sets of fragmentary SNP data of the test-set individuals (Fig. 2).

We denote the number of SNPs within the 1-Mb window around the *ℓ*th Codis locus by *D*_*ℓ*_, with $\mathop {\sum}\nolimits_{\ell=1 }^L {D_\ell } = N_{{{{{{{{\mathrm{win}}}}}}}}}$, and we denote its relative proportion by *p*_*ℓ*_ = *D*_*ℓ*_*/N*_win_. The values of *D*_*ℓ*_ are listed in Table S2. Of *N*_all_ SNPs, the fraction of SNPs present in the *L* windows is *f* = *N*_win_*/N*_all_ ≈ 0.006.

We used a simple model in which distinct SNPs have independent random variables for presence or absence of data. At coverage *c*, the total number of SNPs sequenced is $N_{all}^{\left( c \right)} = \left[ {N_{all}c} \right]$. Assuming all have equal probability of being sequenced, a sequenced SNP lies in one of the *L* 1-Mb windows around the Codis loci with probability *f*. For each simulated fragmentary SNP dataset with coverage *c*, we sampled *X*—a total number of SNPs sequenced in the *L* windows—from a binomial-($N_{all}^{\left( c \right)}$, *f*) distribution. For each test individual *i* in a simulated fragmentary dataset with *X* SNPs sequenced in the *L* windows, we sampled a vector ${{{{{{\boldsymbol{d}}}}}}}^{\left( i \right)} = ( {d_1^{\left( i \right)},\,d_2^{\left( i \right)},...,\,d_L^{\left( i \right)}} )$ from a multinomial-(*X*, *p*) distribution, *p* = (*p*_1_*, p*_2_, ..., *p*_*L*_). Here, $d_\ell ^{\left( i \right)}$ represents a number of SNPs sequenced within the 1-Mb window of the *ℓ*th Codis locus in fragmentary SNP data of individual *i*. For each 1-Mb window around the *ℓ*th Codis locus, we sampled $d_\ell ^{\left( i \right)}$ SNPs uniformly at random without replacement from *D*_*ℓ*_ SNPs in the full-coverage dataset. Figure 2 displays an example.

### Record-matching of STR profiles with fragmentary genomic SNP data

We applied the record-matching pipeline of Section “Genetic record-matching” to each simulated fragmentary SNP dataset of the test-set individuals. As noted in Section “Simulation of fragmentary genomic SNP data,” we fixed the training set at the median-accuracy partition generated in Section “Record-matching with HGDP and 1000 Genomes”; it contained the full-coverage SNP–STR haplotypes of the training-set individuals. For each relatedness scenario, we used a same shared training set across all 100 simulated fragmentary SNP datasets.

We used the training set as a reference in imputing test-set STR profiles from fragmentary SNP profiles according to Eq. (2). We also computed STR allele frequencies from the training set in evaluating Eq. (1).

For the same-individual scenario, the training set contained 1878 individuals and the test set had 626. For each of 100 simulated fragmentary SNP datasets at a given genomic coverage *c*, we computed match scores of all pairs—one with a SNP profile and the other with an STR profile—and obtained a 626 × 626 match-score matrix. We then computed match accuracies under four matching schemes described in Section “Match assignment.” We applied similar procedures for the parent–offspring and sib-pair scenarios (Section S1.6).

## Results

We focus on correctly specified hypotheses, **∆**_true_ = **∆**_test_. Under three relatedness scenarios, we examine the effect of the SNP coverage *c*. This analysis follows the procedures in Section “Record-matching of STR profiles with fragmentary genomic SNP data.” Numerical summaries appear in Table 1. We focus our comments on the same-individual scenario. Results for the parent–offspring and sib-pair analyses are discussed in the supplement (Sections S2.1, S2.2). For completeness, misspecified hypotheses **∆**_true_ ≠ **∆**_test_ also appear in the supplement (Section S2.3, Fig. S1, and Tables S3 and S4).

**Table 1:** Record-matching accuracies using the 1000 Genomes dataset and 15 CODIS loci, for **∆**_true_ = **∆**_test_.

Same individual		Parent-offspring		Sib pairs		Match-assignment scheme
Median	Min, Max	Median	Min, Max	Median	Min, Max	
1.000	1.000, 1.000	0.738	0.681, 0.812	0.693	0.623, 0.773	One-to-one
1.000	0.998, 1.000	0.649	0.581, 0.700	0.626	0.546, 0.674	One-to-many: SNP query
1.000	0.998, 1.000	0.649	0.597, 0.719	0.636	0.572, 0.703	One-to-many: STR query
0.992	0.941, 1.000	0.112	0.016, 0.256	0.160	0.019, 0.275	Needle-in-haystack

The table summarizes 100 partitions into training and test sets, applying record-matching to the 1000 Genomes dataset with the full unfragmented data. The STRs used are listed in Table S2.

### Same individual

**Fig. 3:**
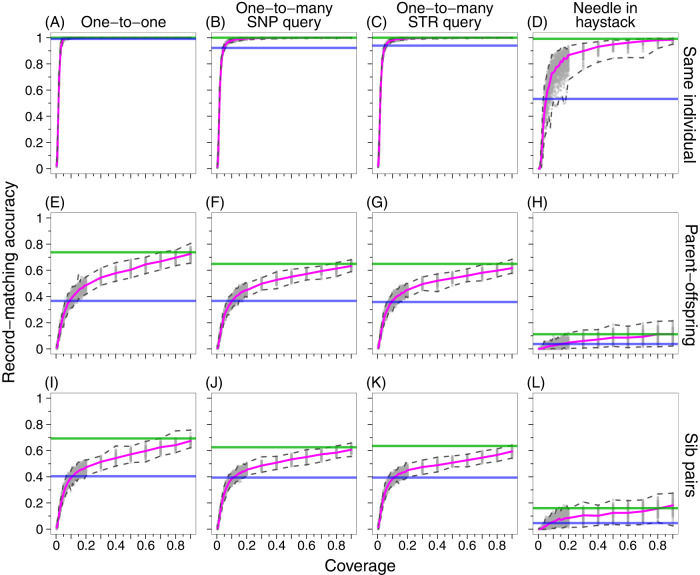
Record-matching accuracy in fragmented genomic data as a fraction of the genomic coverage *c*, for ∆_true_ = ∆_test_. **∆**_true_ is the true relationship between pairs of individuals, and **∆**_test_ is the test relationship hypothesis on the basis of which match scores are computed. **A** Same individual, one-to-one matching. **B** Same individual, one-to-many matching with a query SNP profile. **C** Same individual, one-to-many matching with a query STR profile. **D** Same individual, needle-in-haystack matching. **E** Parent–offspring, one-to-one matching. **F** Parent–offspring, one-to-many matching with a query SNP profile. **G** Parent–offspring, one-to-many matching with a query STR profile. **H** Parent–offspring, needle-in-haystack matching. **I** Sib pairs, one-to-one matching. **J** Sib pairs, one-to-many matching with a query SNP profile. **K** Sib pairs, one-to-many matching with a query STR profile. **L** Sib pairs, needle-in-haystack matching. At each value of *c*, 100 fragmented genomic datasets are considered (Section “Simulation of fragmentary genomic SNP data”). All rely on the same partition of the 1000 Genomes dataset into a test set with 75% of the individuals and a target set with the other 25% (Section “Record-matching with HGDP and 1000 Genomes”); this partition corresponds to the median record-matching accuracy under one-to-one matching with the correctly specified hypothesis (Table 1). The pink line indicates the median of 100 trials with different fragmented datasets; the dashed lines around the pink line specify the minimum and maximum. The green and blue horizontal lines indicate the median record-matching accuracy using the full-coverage 1000 Genomes (Table 1) and HGDP datasets (Table S3), respectively.

Figure 3A–D shows the record-matching accuracy for **∆**_true_ = **∆**_test_ = same individual. For each of four matching schemes, the HGDP median accuracy across partitions produces slightly greater values than in corresponding analyses in Table 2 of our previous study [17], which used 13 rather than 15 loci.

**Table 2:** The fraction of true matches with match score exceeding the minimum threshold for achieving a desired ratio of posterior and prior odds.

	Ratio of posterior odds and prior odds																	
	10^0^	10^1^	10^2^	10^3^	10^4^	10^5^	10^6^	10^7^	10^8^	10^9^	10^10^	10^11^	10^12^	10^13^	10^14^	10^15^	10^16^	10^17^
	Minimum match score																	
	0	2.30	4.61	6.91	9.21	11.51	13.82	16.12	18.42	20.72	23.03	25.33	27.63	29.93	32.24	34.54	36.84	39.14
	Fraction of true matches exceeding the minimum match score																	
Same individual	0.99	0.99	0.98	0.96	0.95	0.93	0.89	0.85	0.81	0.75	0.67	0.58	0.47	0.37	0.27	0.19	0.12	0.09
Parent-offspring	0.88	0.74	0.58	0.35	0.18	0.05	0.02	0.00	0.00	0.00	0.00	0.00	0.00	0.00	0.00	0.00	0.00	0.00
Sib pairs	0.90	0.74	0.53	0.33	0.18	0.07	0.03	0.01	0.00	0.00	0.00	0.00	0.00	0.00	0.00	0.00	0.00	0.00

Note that the ratio of posterior and prior odds is the likelihood ratio for the hypothesis **∆**_test_ in relation to the null hypothesis that two profiles are unrelated. The values correspond to those plotted in Fig. S2.

For all four matching schemes, the median accuracy for the larger and denser 1000 Genomes exceeds that for HGDP; numerical values for HGDP appear in Table S3 and for 1000 Genomes in Table 1 and S4. As the coverage of 1000 Genomes decreases in fragmentary datasets starting from *c* = 0.9, accuracy decreases as well.

For one-to-one matching, decreasing the 1000 Genomes coverage *c* from 0.9, the median accuracy across 100 fragmentary SNP replicates begins at 1 at *c* = 0.9, remaining equal to 1 until coverage *c* = 0.06, for which it drops to 0.997 (Fig. 3A). The HGDP median of 0.991 is achieved in 1000 Genomes at *c* ≈ 0.05. Accuracy drops quickly after *c* = 0.03, with median 0.906; it is 0.677 at *c* = 0.02 and 0.181 at *c* = 0.01.

For one-to-many matching with a SNP query (Fig. 3B) or STR query (Fig. 3C), median accuracy drops somewhat faster than for one-to-one matching. Near ~50% coverage (*c* = 0.5), it drops below 1, though it remains high at much lower coverage. The HGDP median accuracies (0.922, 0.940) are achieved at *c* ≈ 0.05.

For the needle-in-haystack scheme (Fig. 3D), the median accuracy is still lower. The value drops below 0.9 at *c* ≈ 0.3. The HGDP median accuracy for this scheme (0.532) is achieved at *c* ≈ 0.05.

### Ratio of posterior and prior odds

Using Eq. (3), Table 2 and Fig. S2A display the minimum match score *λ* required to achieve a desired ratio of posterior to prior odds, the likelihood ratio. For example, to obtain posterior odds of a match equal to 10^4^ with prior odds 10^−9^, *λ* (Eq. 1) must reach the threshold for likelihood ratio 10^13^, or 29.93.

For each relatedness scenario and a ratio of posterior and prior odds, we computed the fraction of true matches with match score above the minimum (Figure S2A) required for achieving a prescribed ratio. When **∆**_true_ = **∆**_test_ = same individual (Fig. S2B), if the prior probability of two individuals being unrelated is 10^10^ times that of them being the same individual (prior odds 10^−10^), then 67% of true matches achieve posterior odds above 1 (ratio 10^10^), and 9% achieve posterior odds above 10^7^ (ratio 10^17^). When the prior is uninformative, with prior odds of 1, 99% of true matches exceed the match score required for attaining posterior odds of 1 (ratio 10^0^), and 85% of true match pairs have posterior odds above 10^7^ (ratio 10^7^).

## Discussion

We have examined genetic SNP–STR record-matching on fragmentary SNP datasets. For the sequenced genomes of the 1000 Genomes, record-matching accuracies exceed those seen previously in HGDP genotyping arrays (Fig. 3). Accuracies at the level observed for arrays can be obtained in genome sequencing with incomplete coverage, often 5–10% of the genome (Fig. 3). When matching profiles from the same individual, accuracy with the full genome is high in each of four matching schemes—and with one-to-one matching, the record-matching accuracy seen with the full genome is obtained with genomic coverage as low as 6%.

The prior odds value is chosen based on the size of a search population; in a calculation aiming to simulate if a true match could be detected in the United States adult population at posterior probability $\frac{{10}}{{11}}$, Edge et al. [16] found that with 17-locus profiles, 8% of SNP–STR profile pairs matched closely enough that the true match would be detected at likelihood ratio threshold 2.3 × 10^9^. Here, even with 15-locus profiles, 67% of pairs would be detected at a more stringent 10^10^ threshold (Table 2). This result indicates a sizeable probability that a true match of interest could be identified by record-matching with high confidence by a query of a SNP database with an STR profile, or vice versa, even in a large population.

**Fig. 4:**
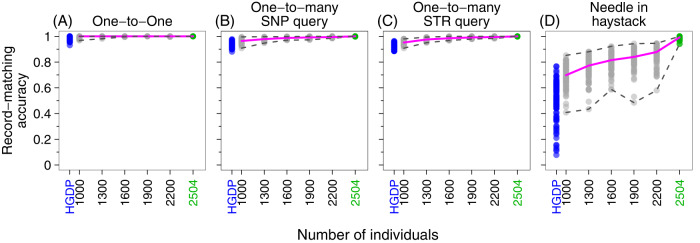
Record-matching accuracy in subsamples of varying size. The figure uses sampled individuals from the 1000 Genomes with full coverage (*c* = 1), considering 15 Codis loci and **∆**_true_ = **∆**_test_ = same individual. For each number of individuals in {1000, 1300, 1600, 1900, 2200}, we randomly sampled 100 sets of individuals from 2504 individuals in the 1000 Genomes dataset and performed record-matching on the reduced dataset, choosing 75% of the individuals for the training set and 25% for the test set. Green points consider all 2504 individuals in the 1000 Genomes and show the values for the 100 replicates summarized in Table 1. **A** One-to-one matching. **B** One-to-many matching with a query SNP profile. **C** One-to-many matching with a query STR profile. **D** Needle-in-haystack matching. The pink line indicates the median one-to-one matching accuracy of 100 trials. For comparison, the blue points indicate the corresponding results using the full HGDP dataset of 872 individuals, reporting the values for the 100 replicates summarized in the upper left corner of Table S3.

The increase in accuracy arises from multiple factors that can improve imputation and in turn, record-matching. First, SNP density in the sequenced 1000 Genomes greatly exceeds that of the earlier HGDP SNP-genotyping studies [16, 17]. Second, the 1000 Genomes has more individuals. Indeed, in an additional analysis of subsamples of the 1000 Genomes, considering full genomic coverage (*c* = 1) and searching for same-individual matches, particularly for the needle-in-haystack scheme, record-matching accuracy increases with sample size (Fig. 4). Hence, enlarging the reference panel to improve the estimation of genotype probabilities in Eq. 2 (“improving the needle detector”) may have a large enough effect in increasing record-matching accuracy to counteract the increase in the number of pairs among which matches are sought (“enlarging the haystack”).

We note that we did not distinguish profiles by source population, considering the entire reference panel as one group. This choice likely decreases record-matching accuracy compared to a potential analysis that would take source populations into account. In particular, conducting record-matching separately in different populations by relying on relevant reference panels for imputation in different subgroups [22–24] could increase imputation accuracy of STRs from SNPs—and by consequence, the record-matching accuracy.

The 1000 Genomes dataset, while larger than the HGDP dataset, relies on imputation of STRs based on an additional (family-based) reference panel; imputation accuracies are high in some 1000 Genomes samples in which direct genotypes are available [[18], Supplementary Table 3]. However, imputation errors occurring during the construction of the 1000 Genomes dataset by Saini et al. [18] might have been replicated in our imputations; our analysis would regard such cases as accurate imputations due to concordance with Saini et al. [18]. While such errors are unlikely to affect the qualitative pattern of imputation accuracy in relation to coverage *c*, in future studies, it will be important to use panels based on SNPs and STRs obtained directly.

Another limitation is that we used a simple simulation to produce fragmentary SNP datasets, assuming that given coverage *c*, SNPs amplify independently, that amplification patterns are independent across individuals, and that genotypes are accurately obtained. With actual degraded DNA, fragmentary datasets likely possess spatial correlation across the genome, containing multiple neighboring SNPs genotyped on the same DNA fragments (Fig. 1B). Inclusion of spatial correlation would increase the probability that given *c*, in some individuals some STRs would possess no neighboring genotyped SNPs—and no information for imputing those STR genotypes. Hence, high levels of spatial correlation in amplification for a fixed coverage could reduce record-matching accuracy, especially at coverage levels low enough to eliminate all SNPs around some STRs. The simulation we have considered is a first approximation; as degradation, amplification, and genotyping error patterns differ for different DNA sources, applications in different settings can deepen the model in ways tailored to their associated fragmentary coverage patterns (e.g. gargammel simulation for ancient DNA [25]).

The results have applications in settings in which an investigator would have liked to test STRs for matches against an STR database, but in which STR genotyping was impossible. If samples are degraded so that only SNP genotypes can be obtained—as might occur for older criminal-justice samples, mass disasters, burned material, or ancient DNA—then our approach could be used to test the resulting SNP profile against an STR database. In such cases, genetic record-matching is used simply to overcome the technical challenge of genotyping STRs in degraded material—in existing investigative settings, not by introducing new ones.

Genetic record-matching can also produce new information linkages if investigators or others possess access to both SNP and STR databases [16, 17]. Profiles in different databases could in principle be connected if biomedical or genealogical participants or their close relatives also appear in forensic STR data. Our results increase the potential accuracy for such efforts. The study contributes to emerging work on cross-database linkages of genetic data, with both investigative potential and privacy risks [26, 27]. We previously [16, 17] discussed privacy risks from the linkages between genetic databases—and possibly phenotype databases—enabled by genetic record-matching; even before the 2018 advent of the long-range search method combining genetic and genealogical data, Edge et al. [16] wrote *“Contrary to the view that* *Codis* *genotypes expose no phenotypes, a* *Codis* *profile on a person together with a SNP database—if the person is in the database—in principle may contain all of the phenotypic information that can be reliably predicted from the SNP record. Conversely, participants in biomedical research or personal genomics who have consented to share their SNP genotypes may be subject to a previously unappreciated risk: identification in a forensic STR database.”*

The increased record-matching accuracy that we have detected in a larger, denser dataset than that used by Edge et al. [16] and Kim et al. [17] only magnifies the privacy concern. The potential for employing genetic record-matching to use one type of individual-level information to reveal information of another type enhances both the potential uses of the technique for individual identification in degraded crime-scene samples, ancient samples, and missing-persons and mass-disasters cases—as well as the potential risks that excess information could be revealed, either by an authorized user or by an attacker. Further consideration is needed of the benefits and privacy risks emerging from cross-database linkages involving SNPs and STRs—and phenotypes.

### Supplementary information

Supplemental Material

## Data Availability

The datasets analyzed in the study are taken from refs. [16] and [18] and are available at http://github.com/jk2236/RM_WGS; programs for implementing steps of the analysis can also be obtained at https://github.com/jk2236/RM_WGS.
