# Supplementary material for: Record-matching of STR profiles with fragmentary genomic SNP data
Source: Eur J Hum Genet. 2023 Aug 11;31(11):1283–90. doi: 10.1038/s41431-023-01430-9 (PMC10620386; doi:10.1038/s41431-023-01430-9)
Supplement: Supplementary file 1 — Supplemental Material [file 41431_2023_1430_MOESM1_ESM.pdf]

Supplementary material

S1 Supplementary methods

S1.1 BEAGLE settings for phasing

We used BEAGLE v. 5.0 (Browning & Browning 2007; Browning *et al.* 2018) for two purposes: phasing (this section) and imputation (Section S1.5). First, in Section 2.3, we phased the entire HGDP dataset to generate the SNP–STR haplotypes from which we simulated random pedigrees (recall that this phasing step was not needed for the pre-phased 1000 Genomes dataset). In Section 2.4, we also phased unphased genotypes in HGDP training datasets.

For these analyses, we set `iterations=14`, `burnin=6`, `phase-states=280`, and `phase-segment=4.0`. We used BEAGLE default parameters shared between the phasing step and the imputation step in Section S1.5: `ne=1000000`, `err=0.0001`, `window=40.0`, `overlap=4.0`, `seed=-99999`, `step=0.1`, and `nsteps=7`.

S1.2 Four matching schemes

In one-to-one matching, for a given row or a column of matrix M, exactly one pair is selected as a match; we assigned matches via the Hungarian algorithm (Kuhn 1955). In one-to-many matching with a query profile (either STR or SNP), an observation in one dataset might be identified as having multiple relationship matches in the other. When the query profile is STR profile R_j, its proposed match is S_k, where $k = \text{argmax}_{u\in[I]} M_{ju}$. With query SNP profile S_k, its proposed match is R_j, $j = \text{argmax}_{u\in[I]} M_{uk}$.

In needle-in-haystack matching, unlike in the other schemes, a database query is performed to locate a match only for one profile. In this setting, perfect accuracy is achieved when the match scores of all true matches exceed those of all non-matching profiles.

S1.3 Pedigree generation

We drew mating pairs uniformly at random without replacement (ignoring population structure); each individual in a dataset appeared in exactly one mating pair. For each mating pair, we simulated two offspring. From each simulated pedigree, we randomly selected one of the parents and one of the offspring siblings for the parent–offspring scenario. We used both siblings for the sib-pair scenario.

In generating offspring haplotypes from parental haplotypes, following Kim *et al.* (2018), we considered 1-Mb SNP windows extending 500 kb in each direction from a Codis locus midpoint. We assumed that our window size was small enough to disregard the possibility of recombination within windows (an event that at

1 cM/Mb has probability 1% per window in a parent–offspring transmission). We also assumed independent assortment of Codis loci (disregarding linkage that might affect some pairs, O'Connor and Tillmar (2012)).

Once pedigrees were generated, we randomized allele orders within individuals, discarding phase information, as the step in which we compute match scores in Section 2.2.1 begins with unphased data.

Note that prior to pedigree generation, for the HGDP dataset, which is unphased, we first used `BEAGLE` to phase the entire HGDP dataset to obtain individual haplotypes (Section S1.1). We then generated 10 random sets of 436 pedigrees from the 872 HGDP individuals.

The 1000 Genomes dataset contains phased haplotypes, so the initial phasing step prior to pedigree generation was omitted. Using 2,504 individuals in the 1000 Genomes dataset, we generated 10 random sets of pedigrees, each with 1,252 simulated pedigrees.

S1.4 Training and test sets

For the same-individual scenario, we generated 100 random partitions into training and test sets. When using the HGDP dataset, each partition contained 654 individuals in the training set and 218 in the test set. For 1000 Genomes, each partition contained 1,878 individuals in the training set and 626 in the test set. We estimated STR allele frequencies from the individuals in the training set.

For the parent–offspring scenario, for each of the 10 random sets of pedigrees, we generated 10 random partitions of the pedigrees into training and test sets, resulting in 100 random partitions in total. In each partition, the numbers of pedigrees in the training and test sets were 327 and 109, respectively, for the HGDP dataset, and 939 and 313 for 1000 Genomes. For each test-set pedigree, without loss of generality, we assigned a SNP profile of a parent to the SNP dataset and an STR profile of an offspring to the STR dataset.

For the sib-pair scenario, we generated 100 random partitions of pedigrees into training and test sets—10 replicates for each of the 10 random pedigree sets. For each test-set pedigree, we randomly placed one sibling in the SNP dataset and the other in the STR dataset. In both parent–offspring and sib-pair scenarios, we estimated STR allele frequencies from the parents in training-set pedigrees.

S1.5 `BEAGLE` settings for imputation

We also used `BEAGLE` v. 5.0 for estimating the unobserved STR genotype probabilities, $\mathbb{P}(R_{B\ell} \mid S_{B\ell})$ in Eq. 2, as in Section 2.2.1. This analysis, which we employed in Sections 2.4 and 2.6, used the phased training-set SNP–STR haplotypes as a reference and augmented them with the SNP genotypes of the unphased test set. We then estimated the genotype probabilities at the STR loci in the test set based on the neighboring SNPs.

This analysis used `BEAGLE` settings matching those used in Section S1.1, except that it used `gp=true`, `impute=true`, `imp-states=1600`, `imp-segment=6.0`, `cluster=0.005`, and `ap=false`. It employed the human reference genome GRCh37 genetic map, whereas the phasing analysis did not use a genetic map.

S1.6 Record-matching for parent–offspring and sib-pair scenarios

For the parent–offspring scenario, the training set contained 1,878 individuals: all parental pairs of the 939 pedigrees in the training set for the median-accuracy partition with $\boldsymbol{\Delta}_{\text{true}} = \boldsymbol{\Delta}_{\text{test}} =$ parent–offspring. The corresponding median-accuracy test set contained the remaining 313 pedigrees (Section 2.4). For each of the 100 simulated fragmentary SNP datasets at genomic coverage c, we computed a 313×313 match-score matrix to match the fragmentary SNP profiles of offspring to the STR profiles of parents in the test set.

Finally, for sib pairs, the training set contained 1,878 individuals, all parental pairs of the 939 pedigrees in the training set of the median-accuracy partition with $\boldsymbol{\Delta}_{\text{true}} = \boldsymbol{\Delta}_{\text{test}} =$ sib pairs. The corresponding median-accuracy test set consisted of the remaining 313 pedigrees (Section 2.4). For each of the 100 simulated fragmentary SNP datasets at genomic coverage c, we computed a 313×313 match-score matrix to match fragmentary SNP profiles to sibling STR profiles in the test set.

S2 Supplementary results

S2.1 Parent–offspring

When $\boldsymbol{\Delta}_{\text{true}} = \boldsymbol{\Delta}_{\text{test}} =$ parent–offspring (Figure 3E-H), accuracies are lower than for the case in which SNP and STR profiles represent the same individual (Figure 3A-D). For all coverage levels $c < 1$, the median accuracy of one-to-one matching (pink line in Figure 3E) is lower than the match accuracy with the full dataset (0.738; green horizontal line). At $c = 0.9$, median accuracy is 0.725; it decreases to achieve the HGDP median one-to-one accuracy of 0.367 (blue horizontal line) at $c \approx 0.09$. At $c = 0.01$, median accuracy is 0.032.

For one-to-many matching with a SNP query (Figure 3F) and one-to-many matching with an STR query (Figure 3G), the median accuracy drops faster from 0.649 and 0.649 at full coverage to 0.374 and 0.371 at $c = 0.1$. The HGDP median values (0.367, 0.358) are achieved at $c \approx 0.1$.

For the needle-in-haystack scheme (Figure 3H), the median accuracy is below the full-coverage median accuracy for all coverage values, dropping from 0.113 at $c = 0.9$ to 0.026 at $c = 0.1$. The HGDP median needle-in-haystack accuracy (0.037) is achieved at $c \approx 0.15$.

For the ratio of posterior and prior odds, When $\boldsymbol{\Delta}_{\text{true}} = \boldsymbol{\Delta}_{\text{test}} =$ parent–offspring (Figure S2C), the

behavior is similar to the same-individual case, but with lower probabilities of attaining specified thresholds. A high value of 88% of true matches achieve posterior odds 1 with prior odds of 1 (ratio 10^0). However, in contrast to the same-individual case, posterior odds values in the range of $[10^0, 10^{11}]$ are largely unattainable with prior odds below 10^{-6} (ratios 10^6 to 10^{17}).

S2.2 Sib pairs

When $\mathbf{\Delta}_{\text{true}} = \mathbf{\Delta}_{\text{test}}$ = sib pairs (Figure 3I-L), accuracies are comparable to those seen when the SNP and STR profiles represent a parent and offspring (Figure 3E-H) and lower than those in which they represent the same individual (Figure 3A-D). For all values of the coverage c, the median accuracy of one-to-one matching (Figure 3I) is lower than the accuracy from the full-coverage data (0.693; green horizontal line). At $c = 0.9$, the one-to-one median accuracy is 0.674, decreasing to achieve the HGDP median one-to-one accuracy (0.404; blue horizontal line) at $c \approx 0.11$. At $c = 0.01$, the median accuracy is 0.038.

For one-to-many matching with a SNP query (Figure 3J) or STR query (Figure 3K), the median accuracy drops from a lower starting point, decreasing from 0.626 and 0.636 at full coverage to 0.383 and 0.383 at $c = 0.1$. The HGDP median one-to-many accuracies (0.394, 0.394) are achieved at $c \approx 0.11$.

For the needle-in-haystack scheme (Figure 3L), the median accuracy lies below the full-coverage median accuracy for all coverage values we simulated, declining from 0.182 at $c = 0.9$ to 0.058 at $c = 0.1$. The HGDP median for the needle-in-haystack scheme (0.046) is achieved at $c \approx 0.08$.

For the ratio of posterior and prior odds, when $\mathbf{\Delta}_{\text{true}} = \mathbf{\Delta}_{\text{test}}$ = sib pairs (Figure S2D), patterns are similar to the parent–offspring case.

S2.3 Misspecified hypotheses $\mathbf{\Delta}_{\text{true}} \neq \mathbf{\Delta}_{\text{test}}$

Figure S1 and Table S4 examine the record-matching accuracies for six pairs of misspecified relatedness hypotheses, $\mathbf{\Delta}_{\text{true}} \neq \mathbf{\Delta}_{\text{test}}$. Accuracies are generally smaller for the misspecified hypotheses than for the correctly specified hypotheses. When profiles represent the same individual ($\mathbf{\Delta}_{\text{true}}$ = same individual), the misspecified parent–offspring ($\mathbf{\Delta}_{\text{test}}$ = parent–offspring) and sib-pair hypotheses ($\mathbf{\Delta}_{\text{test}}$ = sib pairs) continue to detect the relationship with relatively high accuracy. Misspecifying a parent–offspring pair ($\mathbf{\Delta}_{\text{true}}$ = parent–offspring) as sibs ($\mathbf{\Delta}_{\text{test}}$ = sib pairs) or a sib pair ($\mathbf{\Delta}_{\text{true}}$ = sib pairs) as parent–offspring ($\mathbf{\Delta}_{\text{test}}$ = same individual) has a smaller effect on record-matching accuracy than misspecifying either type of pair ($\mathbf{\Delta}_{\text{true}}$ = parent–offspring, $\mathbf{\Delta}_{\text{true}}$ = sib pairs) as arising from the same individual ($\mathbf{\Delta}_{\text{test}}$ = same individual).

Supplementary references

Browning SR, Browning BL, 2007. Rapid and accurate haplotype phasing and missing-data inference for whole-genome association studies by use of localized haplotype clustering. *American Journal of Human Genetics* 81: 1084–1097.

Browning BL, Zhou Y, Browning SR, 2018. A one-penny imputed genome from next-generation reference panels. *American Journal of Human Genetics* 103: 338–348.

Edge MD, Algee-Hewitt BFB, Pemberton TJ, Li JZ, Rosenberg NA, 2017. Linkage disequilibrium matches forensic genetic records to disjoint genomic marker sets. *Proceedings of the National Academy of Sciences* 114: 5671–5676.

Kim J, Edge MD, Algee-Hewitt BF, Li JZ, Rosenberg NA, 2018. Statistical detection of relatives typed with disjoint forensic and biomedical loci. *Cell* 175: 848–858.e6.

Kuhn HW, 1955. The Hungarian method for the assignment problem. *Naval Research Logistics Quarterly* 2: 83–97.

O'Connor KL, Tillmar AO, 2012. Effect of linkage between vWA and D12S391 in kinship analysis. *Forensic Science International: Genetics* 6: 840-844.

Saini S, Mitra I, Mousavi N, Fotsing SF, Gymrek M, 2018. A reference haplotype panel for genome-wide imputation of short tandem repeats. *Nature Communications* 9: 4397.

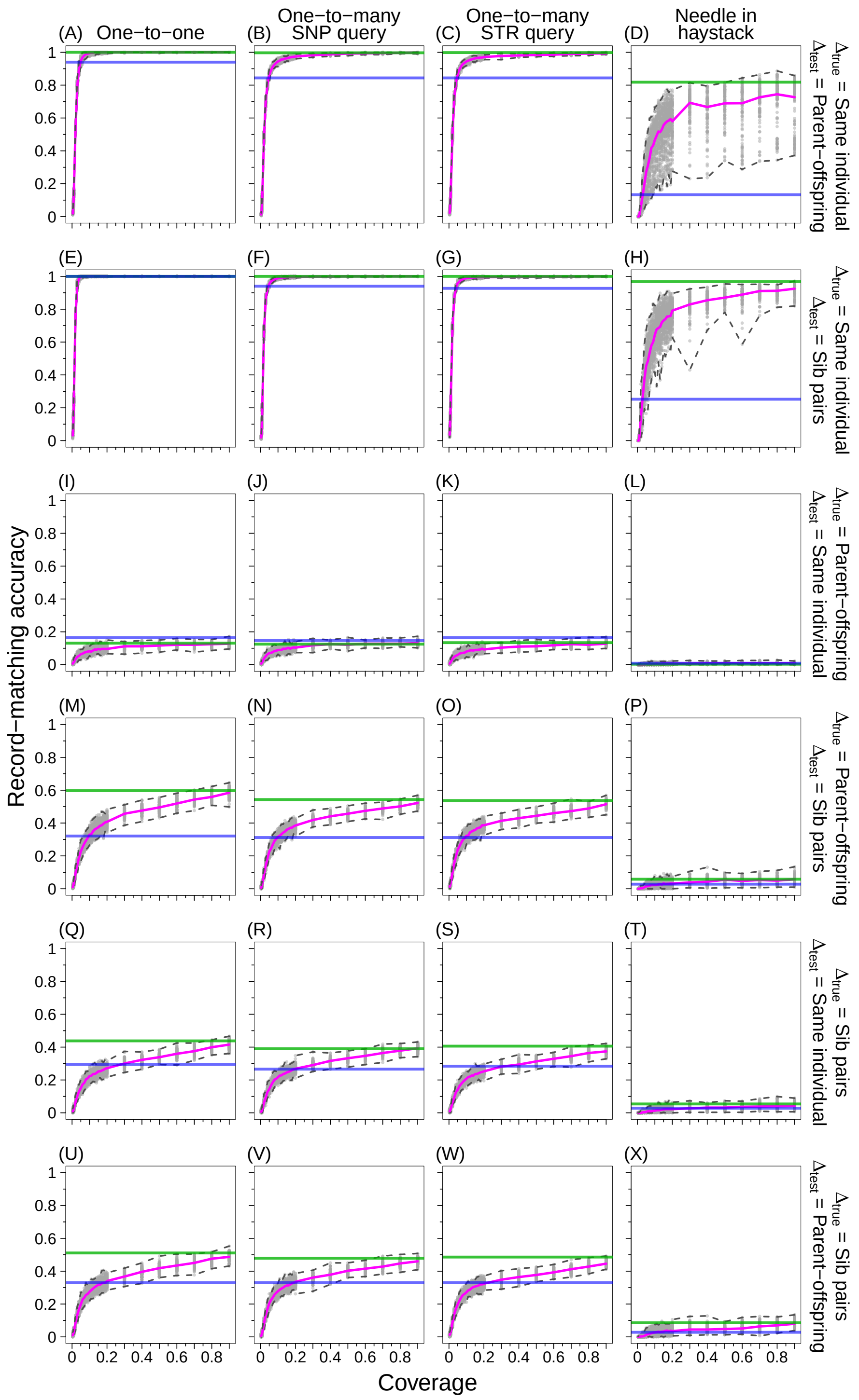

Figure S1: **Record-matching accuracy in fragmented genomic data as a fraction of the genomic coverage c, for $\boldsymbol{\Delta}_{\mathbf{true}} \neq \boldsymbol{\Delta}_{\mathbf{test}}$.** (A-D): $\boldsymbol{\Delta}_{\text{true}}$ = same individual, $\boldsymbol{\Delta}_{\text{test}}$ = parent–offspring. (E-H): $\boldsymbol{\Delta}_{\text{true}}$ = same individual, $\boldsymbol{\Delta}_{\text{test}}$ = sib pairs. (I-L): $\boldsymbol{\Delta}_{\text{true}}$ = parent–offspring, $\boldsymbol{\Delta}_{\text{test}}$ = same individual. (M-P): $\boldsymbol{\Delta}_{\text{true}}$ = parent–offspring, $\boldsymbol{\Delta}_{\text{test}}$ = sib pairs. (Q-T): $\boldsymbol{\Delta}_{\text{true}}$ = sib pairs, $\boldsymbol{\Delta}_{\text{test}}$ = same individual. (U-X): $\boldsymbol{\Delta}_{\text{true}}$ = sib pairs, $\boldsymbol{\Delta}_{\text{test}}$ = parent–offspring. The figure design follows Figure 3.

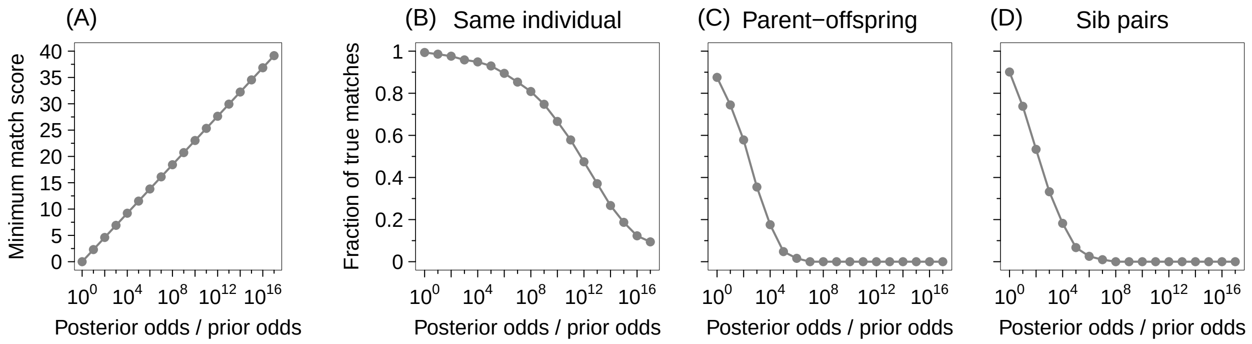

Figure S2: **Numerical values of the fraction of true matches with match score exceeding the minimum threshold for achieving a desired ratio of posterior and prior odds.** (A) The minimum match score required to achieve a desired ratio of posterior and prior odds (Eq. 3). The ratio of posterior and prior odds is the likelihood ratio for hypothesis $\boldsymbol{\Delta}_{\text{test}}$ in relation to the null hypothesis that two profiles are unrelated. (B)–(D) The fraction of true matches with match score exceeding the minimum match score required for achieving a desired ratio of posterior and prior odds (shown in panel A) when $\boldsymbol{\Delta}_{\text{true}} = \boldsymbol{\Delta}_{\text{test}}$ and $c = 1$. The values (Table 2) were computed from 626 individuals (or 313 for parent–offspring and sib pairs) in the test set of the partition corresponding to the median one-to-one match accuracy under full genomic SNP coverage using the 1000 Genomes dataset (Section 2.4 and Table 1). For each value of the ratio of posterior and prior odds and its corresponding minimum match score (panel A), the fraction of true matches was computed as the ratio of the number of true matches with match scores above the threshold and the number of pairs of true matches, 626 (or 313).

**Feature**	**Human Genome Diversity Panel (HGDP)**	**1000 Genomes Project**
**Reference**	Edge *et al.* (2017); Kim *et al.* (2018)	Saini *et al.* (2018)
**Number of individuals**	872	2,504
**Number of populations**	52	26
**Number of SNPs**	642,563	27,185,239
**Number of CODIS STRs included among the original 13 loci**	13	11
**Number of CODIS STRs included among the 7 loci added in 2017**	4	7
**SNP typing approach**	SNP array	Genome sequence
**STR typing approach**	PCR-based	Computational inference

Table S1: **Summary of the features of two datasets used for joint analysis of CODIS STRs and genomic SNPs.**

	Locus	HGDP	1000 Genomes
**13 original CODIS STRs**	**CSF1PO**	334	9,905
	**D13S317**	164	9,253
	**D16S539**	655	NA
	**D18S51**	292	9,510
	**D21S11**	272	NA
	**D3S1358**	300	9,427
	**D5S818**	236	9,170
	**D7S820**	223	9,569
	**D8S1179**	294	9,994
	**FGA**	229	9,360
	**TH01**	254	11,990
	**TPOX**	241	18,330
	**vWA**	325	11,177
**7 CODIS STRs added in 2017**	**D1S1656**	NA	10,618
	**D2S441**	307	10,363
	**D2S1338**	NA	9,843
	**D10S1248**	306	12,021
	**D12S391**	NA	10,243
	**D19S433**	250	10,617
	**D22S1045**	374	11,282

Table S2: **Number of SNPs in 1-Mb windows centered at the CODIS loci in the HGDP and 1000 Genomes datasets.** The 15 STR loci used in our simulations are those loci for which data were present in both the HGDP and 1000 Genomes datasets.

Δ_{test} \ Δ_{true}	Same individual		Parent–offspring		Sib pairs		Match-assignment scheme
	Median	Min, Max	Median	Min, Max	Median	Min, Max	
Same individual	0.991	0.945, 1.000	0.940	0.858, 0.991	1.000	0.963, 1.000	One-to-one
	0.922	0.885, 0.972	0.844	0.789, 0.899	0.940	0.881, 0.972	One-to-many: SNP query
	0.940	0.899, 0.982	0.844	0.775, 0.894	0.927	0.881, 0.972	One-to-many: STR query
	0.532	0.055, 0.803	0.133	0.005, 0.358	0.252	0.046, 0.596	Needle-in-haystack
Parent-offspring	0.165	0.064, 0.248	0.367	0.266, 0.495	0.321	0.220, 0.422	One-to-one
	0.147	0.083, 0.229	0.367	0.220, 0.459	0.312	0.211, 0.404	One-to-many: SNP query
	0.165	0.092, 0.248	0.358	0.257, 0.450	0.312	0.202, 0.431	One-to-many: STR query
	0.009	0.000, 0.037	0.037	0.000, 0.138	0.028	0.000, 0.128	Needle-in-haystack
Sib pairs	0.294	0.156, 0.440	0.330	0.229, 0.450	0.404	0.284, 0.550	One-to-one
	0.266	0.174, 0.358	0.330	0.211, 0.431	0.394	0.294, 0.505	One-to-many: SNP query
	0.284	0.193, 0.385	0.330	0.202, 0.459	0.394	0.275, 0.495	One-to-many: STR query
	0.028	0.000, 0.083	0.028	0.000, 0.119	0.046	0.009, 0.147	Needle-in-haystack

Table S3: **Record-matching accuracies using the HGDP dataset and 15 CODIS loci.** The table summarizes 100 partitions into training and test sets, applying record-matching to the HGDP dataset with the full unfragmented data. The STRs used are listed in Table S2.

$\mathbf{\Delta}_{\text{test}}$ / $\mathbf{\Delta}_{\text{true}}$	Same individual		Parent–offspring		Sib pairs		Match-assignment scheme
	Median	Min, Max	Median	Min, Max	Median	Min, Max	
Same individual	1.000	1.000, 1.000	1.000	1.000, 1.000	1.000	1.000, 1.000	One-to-one
	1.000	0.998, 1.000	0.997	0.990, 1.000	1.000	0.998, 1.000	One-to-many: SNP query
	1.000	0.998, 1.000	0.998	0.994, 1.000	1.000	0.998, 1.000	One-to-many: STR query
	0.992	0.941, 1.000	0.818	0.492, 0.922	0.968	0.898, 0.995	Needle-in-haystack
Parent-offspring	0.131	0.083, 0.204	0.738	0.681, 0.812	0.597	0.514, 0.668	One-to-one
	0.125	0.080, 0.169	0.649	0.581, 0.700	0.543	0.457, 0.613	One-to-many: SNP query
	0.134	0.099, 0.192	0.649	0.597, 0.719	0.537	0.470, 0.610	One-to-many: STR query
	0.003	0.000, 0.032	0.112	0.016, 0.256	0.058	0.003, 0.150	Needle-in-haystack
Sib pairs	0.438	0.361, 0.527	0.511	0.447, 0.585	0.693	0.623, 0.773	One-to-one
	0.390	0.335, 0.470	0.479	0.412, 0.527	0.626	0.546, 0.674	One-to-many: SNP query
	0.406	0.351, 0.476	0.486	0.425, 0.546	0.636	0.572, 0.703	One-to-many: STR query
	0.054	0.010, 0.115	0.086	0.006, 0.169	0.160	0.019, 0.275	Needle-in-haystack

Table S4: **Record-matching accuracies using the 1000 Genomes dataset and 15 CODIS loci.** The table summarizes 100 partitions into training and test sets, applying record-matching to the 1000 Genomes dataset with the full unfragmented data. The STRs used are listed in Table S2. The block diagonal entries with $\mathbf{\Delta}_{\text{true}} = \mathbf{\Delta}_{\text{test}}$ also appear in Table 1.
